# Clinical, radiological and genetic analysis of a male infant with neonatal respiratory distress syndrome

**DOI:** 10.3892/etm.2013.970

**Published:** 2013-02-21

**Authors:** XIAOJUAN YIN, FANPING MENG, WENWEN QU, HANXIAO FAN, LU XIE, ZHICHUN FENG

**Affiliations:** 1 Affiliated Bayi Children’s Hospital, Beijing Military Region General Hospital, Beijing 100700;; 2 Center for Liver Cirrhosis, No. 302 Hospital of PLA, Beijing 100039, P.R. China

**Keywords:** respiratory distress syndrome, surfactant protein B, deficiency, mutation

## Abstract

Surfactant protein B (SP-B) deficiency has become increasingly recognized as a cause of severe prolonged respiratory distress. However, little has been reported with regard to the genetic variability of SP-B in Chinese infants with neonatal respiratory distress syndrome (RDS). One case of a Chinese male infant with neonatal RDS was analyzed for clinical manifestation and genetic variability of SP-B. The clinical manifestations, including grunting, intercostal retractions, nasal flaring, cyanosis and tachypnea were discovered in the physical examination. The initial chest X-ray indicated hyper-inflation, diffuse opacification and air bronchogram of the lungs. Pathological tests of lung tissue revealed RDS and SP-B deficiency. Atelectasis and pneumonedema were observed in the lobes of the lung. Molecular analysis of genomic DNA revealed a mutation of 121del2 in intron 4 of the SP-B gene. In conclusion, the variant in intron 4 of the SP-B gene was associated with neonatal RDS in a Chinese male infant.

## Introduction

Neonatal respiratory distress syndrome (RDS) is a syndrome in premature infants caused by developmental insufficiency of surfactant production and structural immaturity in the lungs. It affects ∼1% of newborn infants and is the leading cause of mortality in preterm infants.

Pulmonary surfactant, a lipoprotein complex, is essential for normal lung function and deficiency of surfactant results in neonatal RDS. A number of studies have suggested a genetic contribution to the etiology of RDS (1,2). Surfactant protein B (SP-B) is important for optimal surfactant function, since it is involved in the pathogenesis of pulmonary disease (3). SP-B is one of the hydrophobic proteins crucial to the surface-lowering properties of surfactant. It is encoded on a relatively small gene of ∼9,500 bp on the short arm of chromosome 2 (4). SP-B deficiency results in severe respiratory failure in term infants shortly after birth and the primary associated diseases are neonatal RDS and acinar dysplasia (5). Since mutations in intron 4 of the SP-B gene are rare, few published studies illustrate the association between mutations in intron 4 of the SP-B gene and various diseases (3,6). To date, there have been no reports of a mutation in intron 4 of the SP-B gene with neonatal RDS in a Chinese population; therefore, we investigated the genetic variability of SP-B intron 4 in an individual with RDS.

## Patient and methods

### Patient

At a gestational age of 42 weeks, the second child of unrelated Chinese parents was delivered by normal vaginal delivery at term (birthweight, 3.98 kg; Apgar score Apgar score of 6 at 1 min and 10 at 5 min). The patient was cyanosed due to respiratory distress aged 1 h and ventilation was initiated. Serial chest radiographs were obtained for assessment of persistent pulmonary hypertension during the course of the disease. The patient was treated in the intensive care unit; however, he failed to respond to treatment and succumbed on day 16. The study was approved by the ethics committee of General Hospital of Beijing Military Region, Beijing, China. Written informed patient consent was obtained from the patient’s family.

### Polymerase chain reaction (PCR) amplification

A 1-ml sample of whole blood was collected in ethylenediamine tetraacetic acid tubes from the proband and a newborn with necrotizing enterocolitis as a control. The genomic DNA of the newborns was purified from the total blood using the Wizard Genomic DNA Purification Kit^®^ (Promega Corporation, Madison, WI, USA) according to manufacturer’s instructions. DNA from the patient and healthy newborn blood samples was amplified by PCR amplification protocols, as described by Gong *et al* (4). The following oligonucleotides were used as PCR primers: 5′-CTGGTCATCGACTACTTCCA-3′; and 5′-TGTGTGTGAGAGTGAGGGTGTAAG-3′.

### Lung tissue biopsy

Formalin-fixed, paraffin-embedded autopsy lung tissue was obtained for immunohistochemical analysis of SP-B protein expression. Antigen retrieval methods were employed initially to ensure that a negative result was due to a lack of protein and not due to a loss of antigenicity. Sections 5 *μ*m thick were cut on a rotary microtome and loaded onto polysine-coated slides. Rabbit polyclonal anti-human SP-B antibody (Abcam, Hong Kong, China) and a streptavidinbiotin complex (SABC) peroxidase elite rabbit IgG kit (Wuhan Boster Biological Technology Ltd., China) were used to detect the antigen-antibody complexes. Sections selected for antigen retrieval were heated in sodium citrate buffer at pH 6.0 for 15 min at 90°C. Endogenous peroxidase was quenched, then sections were blocked with 2% normal goat serum before incubation overnight at 4°C with the primary antibody (dilution, 1:300). After washing with phosphate-buffered saline (PBS), the sections were incubated for 20 min at 37°C with the secondary antibody (goat anti-rabbit IgG marked by biotinylation). Then, the sections were washed with PBS and incubated for 20 min at 37°C with the SABC complexes. Finally, the lung tissue was stained with diaminobenzidine (DAB).

## Results

### Clinical findings

The patient was cyanosed due to respiratory distress aged 1 h and ventilation was initiated. Clinical manifestations, including lividity of the skin, progressive dyspnea with continous groaning, inhaling three concave sign, were discovered in the physical examination. Following conception, the pregnant mother of the proband had no premature rupture, infection or diabetes. The proband had no intrauterine problems or meconium aspiration syndrome during the pregnancy and intrapartum. Full blood count and liver function tests were normal. Blood gas analysis revealed hypercapnemia and hypoxemia, which indicated progressive hypoxic respiratory failure. Routine blood and reactive protein tests were normal. The patient demonstrated pulmonary hypertension and congenital heart disease confirmed by B-mode ultrasonic diagnostic equipment. Serological screenings for enterovirus, adenovirus, influenza types A and B, *Chlamydia trachomatis*, respiratory syncytial virus (RSV), cytomegalovirus (CMV), toxoplasmosis and human immunodeficiency virus (HIV) were all negative. No bacteria was observed by blood culture analysis.

### Radiological findings

**Figure 1:**
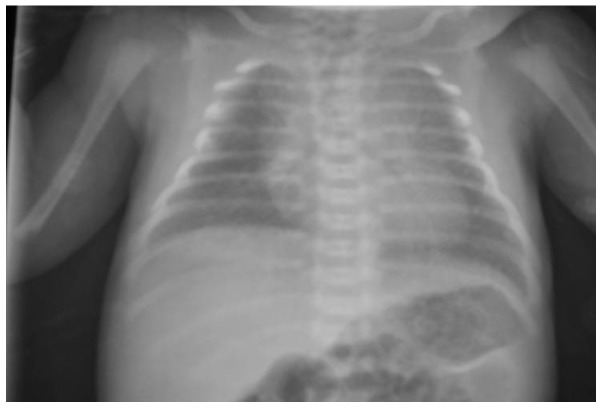
Frontal chest radiograph of the patient aged 1 day revealed hyperinflation, diffuse opacification and air bronchogram of the lungs.

The initial chest X-ray indicated hyper-inflation and diffuse opacification and air bronchogram of the lungs (Fig. 1), which is suggestive of neonatal RDS. A series of therapies, including high-frequency oscillatory ventilation (HFOV), penicillin for preventing infection, intravenous nutrition and oxygen therapy were administered. Serial chest X-rays revealed serious change throughout this period. The pathogenetic condition of the patient worsened and the chest X-ray indicated white lung.

### Genetic findings

**Figure 2:**
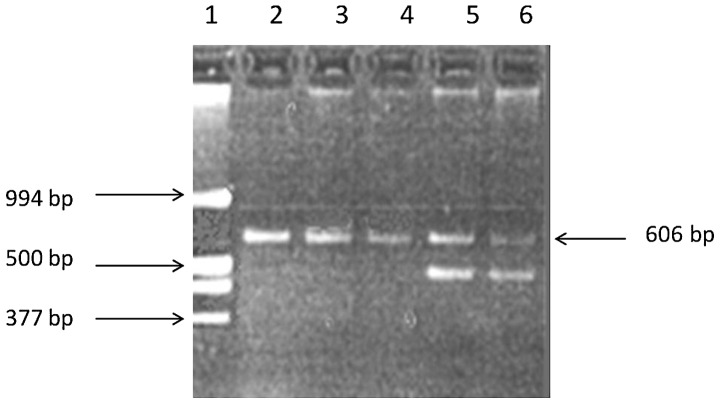
PCR results of the polymorphism in intron 4 of the SP-B gene. Lane 1, the DNA ladder; lanes 2–4, a single 606 bp band is a mild genotype; lanes 5 and 6, the proband with an additional band <606 bp is the deletion variant. PCR, polymerase chain reaction; SP-B, surfactant protein B.

To determine the genetic factors causing RDS, a sample of whole blood was collected for gene analysis. DNA from the patient and healthy newborn blood samples was amplified by PCR amplification protocols. The control with 606 bp bands was considered to be wild-type and the proband with an additional band smaller than 606 bp was considered to have a variant allele, the 121del2 mutation in intron 4 of the SP-B gene (Fig. 2). In this case, the family requested that palliative therapy be administered and the infant succumbed on day 16.

### Pathological findings

**Figure 3:**
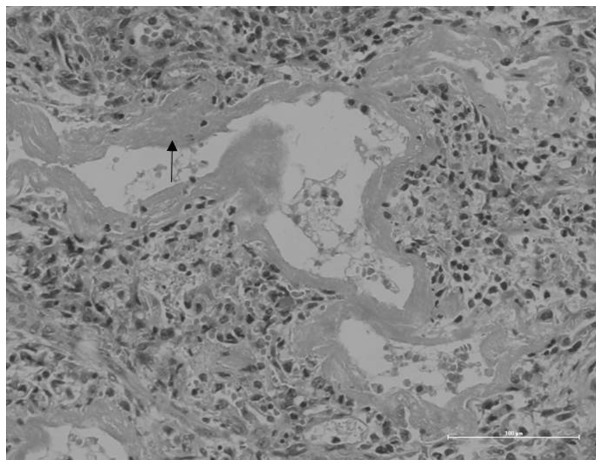
Pathological changes of the lung tissue (hematoxylin and eosin staining, magnification, ×200). High-power magnification shows that the hyaline membrane (arrowhead) sticks to the wall of respiratory bronchioles, alveolar ducts and alveolus.

**Figure 4:**
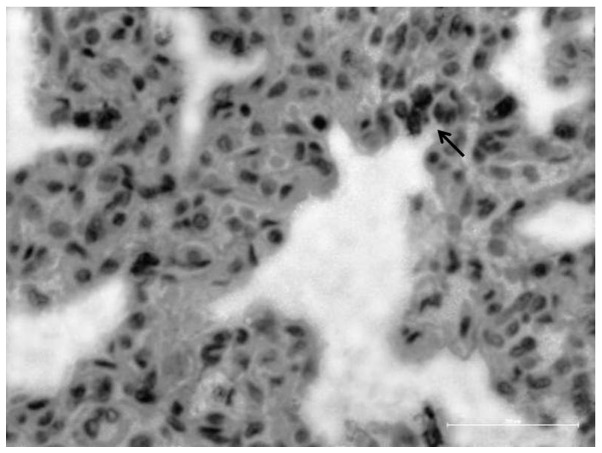
Immunohistochemical analysis of lung tissue for the SP-B protein. (SABC, yellow DAB staining; magnification, ×400). High-power magnification revealed SP-B-positive cells in the lung tissue (arrowhead). SP-B, surfactant protein B; SABC, streptavidin-biotin complex; DAB, diaminobenzidine.

Immunohistochemical characterization and pathological analyses were obtained post-mortem. Pathological changes of the lung tissue were observed with hematoxylin and eosin (HE) staining. This revealed eosinophilic material called the hyaline membrane, which was attached to the wall of the respiratory bronchioles, alveolar ducts and alveolus, and was also observed within the alveolar spaces. The lobes of the lungs had varying degrees of atelectasis and pneumonedema. These manifestations suggest a diagnosis of neonatal RDS (Fig. 3). Our results reveal rarely-expressed SP-B-positive cells in the lung tissue (Fig. 4).

## Discussion

SP-B is essential for maintaining normal surface tension at the air-liquid interface in the alveolus of the lung. The absence of SP-B results in loss of life and dysfunction of SP-B compromises lung function (7,8). Evidence indicates that SP-B exhibits an anti-inflammatory function in the lung (9). The SP-B gene consists of 11 exons (10) and maps on chromosome 2 (11). It encodes a 42 kDa precursor protein (12). The SP-B precursor undergoes several post-translational processing steps to produce a mature protein of 8 kDa, with the last two protein processing steps being type II-cell-specific (13,14).

Neonatal RDS in the newborn is a major cause of neonatal mortality and morbidity. There have been a number of preliminary investigations into the genetic susceptibility of neonatal RDS (1,15). The effect of hereditary factors on neonatal RDS was previously studied. Length variants of intron 4 in the human SP-B gene are associated with several pulmonary diseases. Previous results indicate that intron 4 length variants affect SP-B mRNA splicing and that this may contribute to lung disease (6). Polymorphisms and mutations in the SP-B gene are associated with the pathogenesis of respiratory distress (15). Several studies have demonstrated that deletion variants of intron 4 affect SP-B gene expression (3,4,6); however, SP-B intron 4 variant frequencies have no detectable association with RDS in Brazilian and Finnish populations (1,16). These studies suggest that population, race and the geographical environment affect gene distribution and alignment. In the present study, the association between an SP-B intron 4 variant and neonatal RDS was investigated.

In this study, the patient demonstrated typical clinical, radiological and pathological manifestations of neonatal RDS. The immunohistochemical results of autopsy lung tissue suggested SP-B protein deficiency, and the results of gene analysis indicated that an SP-B intron 4 variant caused SP-B protein deficiency. The onset of progressive hypoxic respiratory failure in the patient was characteristic of RDS. The chest X-ray of the proband revealed hyperinflation, diffuse opacification and air bronchogram in the lungs, indicating a diagnosis of neonatal RDS. The histological appearance was quite dramatic since SP-B-positive cells are rarely observed in lung tissues. Gene analysis revealed that the proband had the 121del2 mutation in SP-B gene intron 4, which is consistent with the study by Gong *et al* (4). The present study suggests the following points: i) the patient had the 121del2 mutation in intron 4 of the SP-B gene; ii) the patient had partial SP-B protein deficiency and iii) the 121del2 mutation in intron 4 of the SP-B gene may cause partial SP-B deficiency, eventually leading to almost irreversible hypoxic respiration. Absence of SP-B may be the most common finding in neonatal RDS; however, it is not identified in all cases. Numerous treatment options have been used for SP-B deficiency; however, treatments involving vigorous surfactant replacement or lavage using cardiopulmonary bypass are unsuccessful. Gene transfer therapy holds future promise for the eventual cure of this usually single gene defect.

In summary, previous studies demonstrated an association between RDS and SP-B gene polymorphism. The polymorphism and mutation of surfactant proteins are helpful for the understanding of the susceptibility to neonatal RDS. However, the association between SP-B gene polymorphism and RDS was previously unclear (17). Our study demonstrates an association between an SP-B intron 4 variant and neonatal RDS in an individual patient. A larger study is required to confirm this finding.
